# Adolescents’ electronic devices use during the COVID-19 pandemic and its relationship to anxiety and depression levels: a cross-sectional study

**DOI:** 10.1186/s12888-023-05482-5

**Published:** 2024-01-10

**Authors:** Suha Hamshari, Shaban Yaseen, Mosab Zayed, Asala Dalasha, Beesan Maraqa, Zaher Nazzal

**Affiliations:** 1 Department of Medicine, Faculty of Medicine and Health Sciences, An-Najah National University, Nablus, Palestine; 2 Faculty of Medicine, Hebron University, Hebron, Palestine

**Keywords:** Adolescents, Anxiety, Depression, Electronic device use

## Abstract

**Background:**

The aim of this study is to assess the prevalence of anxiety and depression symptoms among adolescent students in the West Bank region of Palestine, with a particular focus on the impact of electronic device usage on their mental well-being.

**Methods:**

This cross-sectional study included a representative sample of 1,140 adolescents enrolled in governmental secondary schools. We targeted schools located in Nablus, Ramallah, and Hebron districts, which, respectively, represent the northern, central, and southern regions of the West Bank. We collected data on their sociodemographic characteristics, patterns of electronic device usage, scores from the Beck Depression Inventory-II, and the 7-item Generalized Anxiety Disorder scale, all gathered through a self-administered online questionnaire. To explore the independent relationship between anxiety, depression, and various factors, we calculated odds ratios and their corresponding 95% CI using a binary logistic regression model.

**Results:**

The study revealed a prevalence of moderate to severe anxiety at 35.4% [95% CI: 32.7–38.3%] and moderate to severe depression at 23.9% [95% CI: 21.4–26.4%]. Notably, anxiety scores were significantly higher among females [OR = 3.8, 95% CI: 2.5–5.9], individuals with lower academic performance [OR = 3.4, 95% CI: 2.1–5.4], and smokers [OR = 1.9, 95% CI: 1.1-3.0]. Similarly, significantly elevated depressive scores were observed among females [OR = 2.0, 95% CI: 1.3–3.1], those with lower academic performance [OR = 3.4, 95% CI: 2.1–5.4], and smokers [OR = 1.9, 95% CI: 1.3–2.8]. Conversely, students who used electronic devices for shorter durations were less likely to experience depression [OR = 0.49, 95% CI: 0.32–0.76] or anxiety [OR = 0.47, 95% CI: 0.32–0.69].

**Conclusion:**

Considering the alarming rates of anxiety and depression in adolescents, along with their connection to the time spent using electronic devices, we strongly recommend the creation of initiatives and support networks to alleviate this issue’s impact. Encouraging healthier lifestyles, such as reducing screen time and increasing physical activity, could potentially enhance the mental well-being of adolescents.

**Supplementary Information:**

The online version contains supplementary material available at 10.1186/s12888-023-05482-5.

## Introduction

Adolescence, which spans the ages of 10 to 19, marks the transitional phase between childhood and adulthood and is characterized by numerous physiological, psychological, and social changes [1]. They suffer from various mental disorders [2]; anxiety disorders are characterized by excessive worry and fear that interfere with daily life [3]. Depression is defined as persistent sadness and loss of interest in things that are usually pleasurable for at least two weeks [4, 5]. The global prevalence of mental disorders in this age group was 13.4%; anxiety disorders were 6.5%, and depressive disorders were 2.5% [6].

The COVID-19 pandemic created unprecedented changes in our daily lives, including our physical, economic, and psychological health, resulting in significant disruptions affecting people of all ages [7]. Adolescents were an especially vulnerable group during this period as they faced unique challenges and stressors. During adolescence, interpersonal connections and friendships are vital in shaping their emotional growth [8]. This disconnection became a prominent issue during the pandemic, resulting in increased sleep, reduced physical activity, and greater reliance on electronic devices, all of which contributed to heightened anxiety, further exacerbated by reduced family interaction [9, 10]. The sudden shift to virtual interactions disrupted their social lives, potentially causing them to experience feelings of isolation and loneliness [11]. Adding to this, the pandemic itself was a source of anxiety for many, with concerns about becoming infected, family, and future uncertainties [10, 12].

As a result of the rapid development of technology, electronic devices such as smartphones, tablets, and laptops have become an integral part of our daily lives, especially for students who use them as study tools, reading aids, and other purposes. Increased reliance on electronic devices became a distinguishing feature of the pandemic period as distant learning and social distancing methods became the norm [13], which led to a rise in daily screen time to 4.38 h, up from the pre-pandemic average of 2.67 h [14]. This heightened screen time could potentially have adverse effects on the physical and mental health of adolescents, potentially resulting in problems like back pain, headaches, obesity, sleep disturbances, anxiety, and depression [5, 15–17]. Moreover, 4 h of daily screen time was associated with low curiosity, self-control, and distractibility [18].

Adolescents are particularly vulnerable to maintaining their mental health and require integrated and multidisciplinary services to expand the range of interventions and reduce the risk of a poor long-term outcome [19]. All of these lifestyle changes and the possibility of a prolonged or reoccurring pandemic have raised concerns regarding their potential impact on the mental health of adolescents, highlighting the importance of researching this topic.

Furthermore, Palestine’s unique situation as one of the few countries in the world suffering the occupation and the resulting economic, social, and psychological strains highlight the critical need for research into the mental health of adolescents in this region [20]. This study aims to estimate the prevalence of depression and anxiety among adolescents, as well as the impact of electronic device use on their mental health. The findings of this study have the potential to inform the development of guidelines and recommendations for parents, educators, and policymakers, providing advice on responsible electronic device usage and approaches to improve adolescents’ mental health.

## Methods

### Participants

The present study utilized a cross-sectional design and comprised a representative sample of 1,140 students attending secondary schools. The secondary level of education serves as a bridge between the elementary level and university education. We included students of the tenth and eleventh grades (16 and 17 years old) who were currently enrolled in governmental secondary schools. We excluded the final stage (twelfth grade) because this is the stage that determines the future and destiny of students in Palestine through an exam at the end of the year that allows them to apply to universities and places enormous mental strain on students. The selected schools were located in Nablus, Ramallah, and Hebron districts, which, respectively, represent the northern, central, and southern regions of the West Bank.

Given the total population (N) of 146,206, the geographical regions of the West Bank, North, Middle, and South, and the hypothesized prevalence (p) of 50%, a sample size of 1150 is expected, with an acceptable error of 5% on either side (0.05) and a confidence level of 95%. We randomly selected ten schools from each region of the West Bank, comprising rural and urban, male and female schools, to participate in the study. Following that, we invited all students in the tenth and eleventh grades from the selected schools.

### Measurement tools and procedures

We obtained information from adolescents between February and April 2022 using a self-administered online survey created with Google Forms. Children and adolescents accurately and reliably report their health status. Self-reported health is a valid indicator of several physical and psychological dimensions of adolescent well-being [21, 22], and they preferred the online questionnaire delivery method [23]. The Ministry of Education assigned us three research moderators, one for each region, who managed the distribution of the questionnaire via the official websites of the randomly selected schools.

The questionnaire started with a mandatory question about whether the participant wanted to participate. It was divided into three sections with multiple-choice questions—the first section inquired about residency, gender, grade, field, and academic performance. The Generalized Anxiety Disorder-7 scale (GAD-7) and Beck’s Depression Inventory-II (BDI-II) comprised the second and third sections of the questionnaire.

The GAD-7, which consists of seven self-reported questions, is a screening instrument for anxiety. It had a total score of 21, with 5–9 indicating mild anxiety, 10–14 indicating moderate anxiety, and 15 or more indicating severe anxiety. Using a cutoff score of 10, the GAD-7 has a sensitivity of 89% and a specificity of 82% for identifying generalized anxiety disorder [24]. In this study, we used the translated and validated Arabic version of the GAD-7 [25], and its internal consistency was excellent (0.88 Cronbach’s alpha).

The BDI-II, used for screening the severity of depression in adolescents, consists of 21 items. Each item is rated on a 4-point scale ranging from 0 (symptom not present) to 3 (symptom strongly present), and the cutoff points for mild, moderate, and severe depression are 13, 19, and 28, respectively. Ghareeb AG. examined The psychometric properties of the Arabic version in 17 Arab countries [26]. The validity and reliability of the Beck’s Depression Inventory-II Arabic version have been evaluated across various Arabic-speaking population groups, demonstrating its effectiveness for assessing depression [27, 28]. The BDI-II used in this study demonstrated excellent internal consistency (Cronbach’s alpha = 0.92).

The An-Najah National University Institutional Review Board (IRB) granted ethical approval [Reference #: MED August 2021/18], and the Ministry of Education approved the study’s conduct. Participants were informed about the study’s goal and significance, that participation was voluntary, and that the data would be kept confidential, anonymous, and utilized purely for research purposes. All participants gave an informed consent. According to national law, parents of secondary school students (10th to 12th grades) are not required to sign an additional consent from their parents if the Ministry has approved the study.

### Statistical analysis

The collected data was downloaded as an Excel spreadsheet from a Google form and filtered and coded. Version 23 of the SPSS program (IBM Corp., Armonk, NY: IBM Corp) was utilized for descriptive and inferential statistical analyses, with frequency tables constituting the majority of descriptive analyses. The mean and standard deviation were used to characterize continuous variables (SD). The Chi-square test was used to examine the significance of the association between categorical variables. Using a binary logistic regression model, we calculated odds ratios (ORs) and their 95% confidence intervals (CIs) to examine the independent relationship between anxiety and depression and various independent factors. Our findings and interpretations are based on a 5% level of significance.

## Results

### Background characteristics

This study includes 1140 students in the tenth (49.2%) and eleventh (50.8%) grades from secondary schools in the West Bank region of Palestine. Table 1 shows that the majority (76.5%) were females, and 54.7% were from the northern districts. Academic performance in the previous year was excellent and very good for 46.6% and 27% of students, respectively. Approximately 11% of students smoke, and 90% exercise less than three hours weekly.

**Table 1:** Sociodemographic characteristics of secondary school students in Palestine (n = 1140)

Characteristics	Frequency	Percentage
**Districts**		
North	624	54.7%
Middle	376	33.0%
South	140	12.3%
**Gender**		
Male	267	23.4%
Female	873	76.6%
**Grade**		
10th Grade	561	49.2%
11th Grade	579	50.8%
**The field for 11th -grade students (n = 579)**		
Scientific	350	61.3%
Art	159	27.8%
Others†	62	10.9%
**Academic performance**		
Excellent (90–100)	531	46.6%
Very good (80–89)	308	27.0%
Good (70–79)	185	16.2%
Intermediate or acceptable (< 70)	116	10.2%
**Smoking**		
Yes	122	10.7%
No	1016	89.3%
**Duration of exercise**		
< 1 h per week	657	57.6%
1–2 h per week	272	23.9%
2–3 h per week	97	8.5%
≥ 3 h per week	114	10.0%

†*Others include industrial, agricultural, commercial, and nursing and hotel fields.*

### Electronic devices use

Data on the use of electronic devices (type, purpose, and duration) are displayed in Table 2. Most students (92.9%) use smartphones, 46.5% use laptops, 15.2% use tablets, and 9.6% play video games. The students cited communication (77.5%), entertainment (76.7%), and education (67.5%) as the main reasons for using electronic devices; however, when asked about the most common reasons for using electronic devices, they chose entertainment and communication (41.1%). Almost half (48.4%) of students spent more than three hours a day on their electronic devices, and 23.9% spent two to three hours a day.

**Table 2:** Electronic devices use characteristics

Characteristics	Frequency	Percentage
**Type of electronic device**		
Smartphone	1059	92.9%
Laptop	530	46.5%
Tablet	173	15.2%
Videogames player	110	9.6%
**Purposes of use**		
Communication	884	77.5%
Entertainment	874	76.7%
Education	770	67.5%
**Duration of use (hours/day)**		
< 1 h	111	9.7%
1–2 h	205	18%
2–3 h	273	23.9%
> 3 h	551	48.4%
**Most used device for each student**		
Smartphone	983	86.2%
Laptop	86	7.5%
Tablet	51	4.5%
Videogames players	20	1.8%
**The most reason for using the devices**		
Entertainment	468	41.1%
Education	204	17.9%
Communication	468	41.1%

### Anxiety and depression

**Fig. 1:**
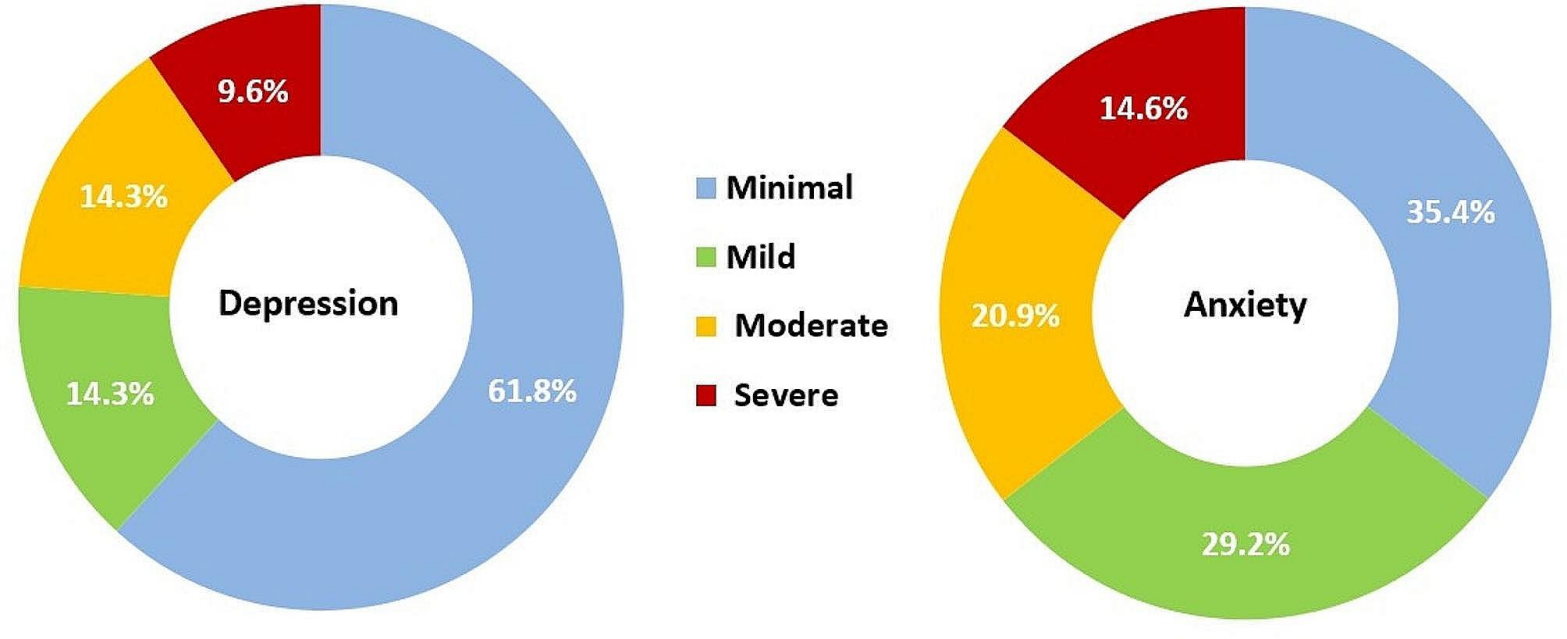
Level of anxiety and depression among adolescents

Supplementary Tables 1 and 2 present adolescents’ responses to the GAD-7 scale and BDI-II items. The GAD-7 scale had an average total score of 7.7 ± 5.7, with scores spanning from 0 to 21. Based on this score, 20.9% experienced moderate anxiety, and 14.6% had severe anxiety. The combined prevalence of moderate to severe anxiety was 35.4% [95% confidence interval: 32.7 − 38.3%]. For the BDI-II, the mean total score was 12.2 ± 10.6, ranging from 0 to 57. Approximately one-third (28.6%) showed mild to moderate depression, and 9.6% experienced severe depression (Fig. 1).

On univariate analysis, district, gender, grade, academic performance, duration of exercise, and duration of device use were found to be associated with anxiety. Multivariable analysis showed that anxiety is more likely among females [P ≤ .001, OR = 3.8 (95%CI:2.5–5.9)], students from south districts [P ≤ .001, OR = 1.8 (95%CI: 1.4–2.3)], 11th -grade students [P ≤ .001, OR = 1.8 (95%CI:1.4–2.3)], students with lower academic performance [P = .001, OR = 1.9 (95%CI:1.3–2.8) and P ≤ .001, OR = 3.2 (95%CI: 2.0–5.0)], smokers[P = .014,OR = 1.9 (95%CI:1.1-3.0)]. On the other hand, students with a lower duration of electronic devices usage [P = .008, OR = 0.53 (95%CI: 0.33-0.85), P ≤ .001, OR = 0.47 (95%CI: 0.32-0.69) and P = .003, OR = 0.61 (95%CI:0.44-0.85)] were less likely to have anxiety (Table 3).

**Table 3:** Results of univariate and multivariate analyses of student characteristics and anxiety

	Normal (n = 736)	Anxiety (n = 404)	P value	adjusted P value	aOR (95%CI)
**District**					
North	428 (68.6%)	196 (31.4%)	0.002	-	-
Middle	225 (59.8%)	151 (40.2%)		0.106	1.3 (0.95 − 1.7)
South	83 (59.3%)	57 (40.7%)		0.001	2.2 (1.4–3.3)
**Gender**					
Male	212 (79.4%)	55 (20.6%)	< 0.001	-	-
Female	524 (60.0%)	349 (40.0%)		< 0.001	3.8 (2.5–5.9)
**Grade**					
10th Grade	401 (71.5%)	160 (28.5%)	< 0.001	-	-
11th Grade	335 (57.9%)	244 (42.1%)		< 0.001	1.8 (1.4–2.3)
**Field**					
Scientific	194 (55.4%)	156 (44.6%)			
Art	100 (62.9%)	59 (37.1%)	0.248		
Others	38 (61.3%)	24 (38.7%)			
**Academic performance**					
Excellent (90–100)	362 (68.2%)	169 (31.8%)		-	-
Very good (80–89)	208 (67.5%)	100 (32.5%)	< 0.001	0.179	1.3 (0.9-1.7)
Good (70–79)	110 (59.5%)	75 (40.5%)		0.001	1.9 (1.3–2.8)
Intermediate or acceptable (< 70)	56 (48.3%)	60 (51.7%)		< 0.001	3.2 (2–5)
**Smoking**					
Yes	73 (59.8%)	49 (40.2%)	0.248	0.014	1.9 (1.1-3)
No	663 (65.1%)	355 (34.9%)		-	-
**Duration of exercise**					
< 1 h per week	397 (60.4%)	260 (39.6%)		0.508	1.2(0.73 − 1.9)
1–2 h per week	188 (69.1%)	84 (30.9%)	0.006	0.743	0.92 (0.55 − 1.5)
2–3 h per week	72 (74.2%)	25 (25.8%)		0.711	0.89 (0.46 − 1.7)
≥ 3 h per week	79 (69.3%)	35 (30.7%)		-	-
**Duration of devices use**					
< 1 h per day	79 (71.2%)	32 (28.8%)		0.008	0.53 (0.33-0.85)
1–2 h per day	155 (75.6%)	50 (24.4%)	< 0.001	< 0.001	0.47 (0.32-0.69)
2–3 h per day	192 (70.3%)	81 (29.7%)		0.003	0.61 (0.44-0.85)
> 3 h per day	310 (56.3%)	240 (43.7%)		-	-

***aOR***: *adjusted Odds Ratio;****CI***: *Confidence Interval*

Univariate analysis of the factors associated with depression revealed that district, smoking, gender, grade, academic performance, and duration of using electronic devices per day were all associated with depression. According to multivariable logistic regression, depression was significantly more likely among students from the middle district [P = .001, OR = 1.7 (95%CI:1.2–2.3)], females [P ≤ .001, OR = 2.0 (95%CI: 1.3–3.1)], students in the11th grade [P = .001, OR = 1.7 (95%CI: 1.3–2.2)], students with lower academic performance [P = .001, OR = 2.0 (95%CI: 1.3-3.0), and P ≤ .001, OR = 3.4 (95%CI:2.1–5.4)], and students who smoked [P = .038, OR = 1.7 (95%CI:1.1–2.8)]. Students who used electronic devices for a shorter period, on the other hand, were less likely to be depressed [P = .001, OR = 0.49 (95%CI: 0.32-0.76) and P = .001, OR = 0.52 (95%CI: 0.35-0.76)] (Table 4).

**Table 4:** Results of univariable and multivariable analyses on student characteristics and depression

	Normal (n = 868)	Depression (n = 272)	P value	adjusted P value	aOR (95%CI)
**District**					
North	499 (80.0%)	125 (20.0%)	0.002	-	-
Middle	264 (70.2%)	112 (29.8%)		0.002	1.7 (1.2–2.3)
South	105 (75.0%)	35 (25.0%)		0.088	1.5 (0.94 − 2.4)
**Gender**					
Male	217 (81.3%)	50 (18.7%)	0.025	-	-
Female	651 (74.6%)	222 (25.4%)		0.003	2 (1.3–3.1)
**Grade**					
10th Grade	453 (80.7%)	108 (19.3%)	0.001	-	-
11th Grade	415 (71.7%)	164 (28.3%)		0.001	1.7 (1.3–2.2)
**Field** (for 11th grade)					
Scientific	248 (70.9%)	102 (29.1%)			
Art	117 (73.6%)	42 (26.4%)	0.810		
Others	45 (72.6%)	17 (27.4%)			
**Academic performance**					
Excellent (90–100)	431 (81.2%)	100 (18.8%)		-	-
Very good (80–89)	233 (75.6%)	75 (24.4%)	< 0.001	0.011	1.6 (1.1–2.3)
Good (70–79)	134 (72.4%)	51 (27.6%)		0.001	2 (1.3-3)
Intermediate or acceptable (< 70)	70 (60.3%)	46 (39.7%)		< 0.001	3.4 (2.1–5.4)
**Smoking**					
Yes	81 (66.4%)	41 (33.6%)	0.008	0.038	1.7 (1.0-2.8)
No	787 (77.3%)	231 (22.7%)		-	-
**Duration of exercise**					
< 1 h per week	492 (74.9%)	165 (25.1%)		0.526	0.85 (0.52 − 1.4)
1–2 h per week	216 (79.4%)	56 (20.6%)	0.493	0.235	0.72 (0.41 − 1.2)
2–3 h per week	75 (77.3%)	22 (22.7%)		0.925	0.99 (0.5-1.9)
> 3 h per week	85 (74.6%)	29 (25.4%)		-	-
**Duration of devices use**					
< 1 h per day	88 (79.3%)	23 (20.7%)		0.064	0.62 (0.37 − 1.0)
1–2 h per day	172 (83.9%)	33 (16.1%)		0.001	0.49 (0.32-0.75)
2–3 h per day	226 (82.8%)	47 (17.2%)	< 0.001	0.001	0.52 (0.35-0.75)
> 3 h per day	382 (69.3%)	169 (30.7%)		-	-

***aOR***: *adjusted Odds Ratio;****CI***: *Confidence Interval;*

## Discussion

Mental health disorders impact an average of 15% of children and adolescents globally [29], with depression and anxiety being the most frequent mental illnesses affecting this age group [6]. According to our research, 35.4% of Palestinian adolescents in the West Bank exhibit moderate to severe anxiety symptoms, while 23.9% exhibit moderate to severe depression symptoms. These figures are lower than those in Gaza strip, where 89.1% have significant anxiety and 72.1% experience severe depression [30]. In Jordan, studies show high levels too: 49.1% and 78.2% of adolescents have severe depressive symptoms and anxiety in one study [31], while another reports 73.8% and 42.1% of students facing depression and anxiety [32]. However, our findings align with certain results documented in the region. For instance, a study conducted in Saudi Arabia involving secondary school students revealed that 35.2% experienced anxiety and 30.8% dealt with depression [33]. Another Saudi study found that 32.4% of participants exhibited signs of depression [34]. Additionally, a population-based study in the UAE indicated that the overall prevalence of anxiety disorders among adolescents was 28.0% [35]. The findings exceed those reported in a recent Greek study, which found that 29.0% and 15.0% of young individuals had symptoms of depression and anxiety, respectively [36].

As numerous factors contribute to adolescent mental health issues, differences in anxiety and depression screening scales, as well as different age groups specified in each study, it is difficult to determine the difference in prevalence. Consequently, further subgroup analyses should be utilized. The literature have shown that the rates of depression and anxiety rose during the COVID-19 pandemic, which corresponds to the period during which our sample was collected, suggesting that this age group is more susceptible to depression and stress symptoms during this time [37–39].

Similar to other studies, we found female adolescents are more likely to have mental health issues than male adolescents; females were more likely to be anxious and depressed. Researchers attributed the higher anxiety levels reported by female adolescents in the Greek study to societal pressures, the role of gonadotropins, life stressors, and psychosocial factors, as reported in other literature [36, 40]. Different environmental and cultural factors may contribute to anxiety disorders in Arab and Muslim nations. Some sociodemographic characteristics, including the father’s education, the mother’s employment status, the siblings’ ranking, and the type of school, had a substantial effect on the mental health of Saudi Arabian adolescents [41]. In Egypt, however, males had a higher prevalence of anxiety disorders due to larger family sizes [42]. In countries such as Palestine, people who had witnessed the violence associated with armed conflict reported PTSD and other anxiety disorders, a phenomenon that is more likely to affect females [43].

Each student’s previous academic performance was correlated with anxiety and depression. The greater an individual’s anxiety and depression, the lower their academic performance. This can be interpreted in two ways. First, the fact that students with higher test scores are happier with themselves and experience positive emotions that their surroundings may enhance, and second, the fact that students with depression and anxiety are less capable of achieving higher grades necessitates an intervention for those identified as depressed or anxious and a subsequent evaluation of their performance.

Smokers are approximately twice as likely to experience depressive symptoms, which may suggest that smoking is a risk factor for depression or that people who are depressed are more likely to smoke because they think it will make them feel better. In other studies, adolescents who smoked showed higher levels of anxiety and depression [36]. Some studies have linked depression to smoking’s neuropharmacology effects on neurotransmitter systems. In addition, smoking is a common self-medication for those who are depressed [44]. Even though this is a self-reported estimate and the quantity and frequency of smoking were not questioned, smoking among adolescents is a significant problem that needs to be addressed in systemic analysis, particularly in light of the development of e-cigarettes [45]. Similarly, research demonstrates that exercise decreases anxiety and depression [46], and longer exercise sessions reduce the prevalence of anxiety in our sample.

Our study showed that the amount of time spent each day using electronics was strongly correlated with anxiety and depression; adolescents who use electronic devices for more than three hours a day are more likely to experience anxiety or depressive symptoms. Adult depression and the timing and duration of electronic device use have been linked [47]. Those who used screens for seven or more hours per day were more than twice as likely to have been diagnosed with depression or anxiety, seen a mental health professional, or taken medication for a mental or behavioral health issue. Even moderate screen time (4 h per day) was associated with poorer mental health [18]. The use of electronic devices by adolescents and excessive screen time has been associated with various negative effects, including disrupted sleep, greater academic distractions, reduced social interactions with friends and family, increased propensity for violence and risk-taking behaviors, limited physical activity, weight gain, depression, and substance abuse [48]. Another point to consider is that during the pandemic, school closure boosted the use of electronic devices since students learned through different online platforms on their electronic devices [49]. This resulted in students spending more time on their devices and being socially isolated [50].

We were not permitted to enter the schools to collect data for our study due to the peak of the COVID-19 quarantine; thus, we used an online questionnaire, and our sample was convenient, which was one of our study’s limitations. Limitations include the cross-sectional design and lack of temporality. Furthermore, the online survey data collection method makes it susceptible to social desirability bias in reporting. Nevertheless, we have a large sample size, limit our age group analysis to two-year intervals, which we consider a strength, and used a highly specific and reliable questionnaire to assess the most significant mental health issue affecting adolescents.

## Conclusion

The findings of this study indicate that a significant proportion of adolescents experience moderate to severe anxiety and depression symptoms. Several factors, including female gender, lower academic performance, smoking, and physical inactivity, were associated with a higher likelihood of anxiety and/or depression. Additionally, the duration of daily electronic device use was significantly correlated with anxiety and depression. These findings suggest that the mental health of Palestinian adolescents is a matter of concern, particularly in the context of pandemics, and emphasize the need to address their mental health needs and develop interventions and support systems to reduce the prevalence of anxiety and depression in this population. Furthermore, strategies to promote healthier lifestyles, such as increased physical activity and reduced screen time, could contribute to better mental well-being among adolescents.

### Electronic supplementary material

Below is the link to the electronic supplementary material.

Supplementary Material 1

Supplementary Material 2

## Data Availability

The datasets generated during and analyzed during the current study are available from the corresponding author on reasonable request.
