# Supplementary material for: Adolescents’ electronic devices use during the COVID-19 pandemic and its relationship to anxiety and depression levels: a cross-sectional study
Source: BMC Psychiatry. 2024 Jan 10;24:38. doi: 10.1186/s12888-023-05482-5 (PMC10777600; doi:10.1186/s12888-023-05482-5)
Supplement: Supplementary file 1 — Supplementary Material 1 [file 12888_2023_5482_MOESM1_ESM.docx]

| **Supplementary Table 1**: Frequency of adolescents’ answers to Generalized Anxiety Disorder-7 scale items. | | | | |
| --- | --- | --- | --- | --- |
|  | **Not at all**  **n (%)** | **Several days**  **n (%)** | **More than half of days**  **n (%)** | **Nearly every day**  **n (%)** |
| Feeling nervous, anxious, or on edge. | 314 (27.5) | 436 (38.2) | 139 (12.2) | 251 (22.0) |
| Not being able to stop or control worrying. | 497 (43.6) | 380 (33.3) | 126 (11.1) | 137 (12.0) |
| Worrying too much about different things. | 334 (29.3) | 466(40.9) | 118 (10.4) | 222 (19.5) |
| Trouble relaxing. | 473 (41.5) | 387 (33.9) | 120 (10.5) | 160 (14.0) |
| Being so restless that it is hard to sit still. | 480 (42.1) | 329 (28.9) | 138 (12.1) | 193 (16.9) |
| Becoming easily annoyed or irritable. | 319 (28.0) | 376 (33.0) | 130 (11.4) | 315 (27.6) |
| Feeling afraid, as if something awful might happen | 528 (46.3) | 338 (29.6) | 119 (10.4) | 155 (13.6) |
