# Supplementary material for: Adolescents’ electronic devices use during the COVID-19 pandemic and its relationship to anxiety and depression levels: a cross-sectional study
Source: BMC Psychiatry. 2024 Jan 10;24:38. doi: 10.1186/s12888-023-05482-5 (PMC10777600; doi:10.1186/s12888-023-05482-5)
Supplement: Supplementary file 2 — Supplementary Material 2 [file 12888_2023_5482_MOESM2_ESM.docx]

| **Supplementary Tables 2**: Frequency of adolescents’ answers to Beck's Depression Inventory items | |  |
| --- | --- | --- |
|  | ***Items*** | **N (%)** |
| ***Sadness*** | I do not feel sad | 620 (54.4) |
|  | I feel sad | 327 (28.7) |
|  | I am sad all the time and I can't snap out of it | 124 (10.9) |
|  | I am so sad and unhappy that I can't stand it | 69 (6.1) |
| ***Pessimism*** | I am not particularly discouraged about the future | 765 (67.1) |
|  | I feel discouraged about the future | 168 (14.7) |
|  | I feel I have nothing to look forward to | 118 (10.4) |
|  | I feel the future is hopeless and that things cannot improve | 89 (7.8) |
| ***Past failure*** | I do not feel like a failure | 838 (73.5) |
|  | I feel I have failed more than the average person | 146 (12.8) |
|  | As I look back on my life, all I can see is a lot of failures | 92 (8.1) |
|  | I feel I am a complete failure as a person | 64 (5.6) |
| **Loss of pleasure** | I get as much satisfaction out of things as I used to | 566 (49.6) |
|  | I don't enjoy things the way I used to | 419 (36.8) |
|  | I don't get real satisfaction out of anything anymore | 102 (8.9) |
|  | I am dissatisfied or bored with everything | 53 (4.6) |
| **Guilty feelings** | I don't feel particularly guilty | 487 (42.7) |
|  | I feel guilty a good part of the time | 477 (41.8) |
|  | I feel quite guilty most of the time | 117 (10.3) |
|  | I feel guilty all of the time | 59 (5.2) |
| **Punishment feelings** | I don't feel I am being punished | 720 (63.2) |
|  | I feel I may be punished | 238 (20.9) |
|  | I expect to be punished | 88 (7.7) |
|  | I feel I am being punished | 94 (8.2) |
| ***Self-dislike*** | I don't feel disappointed in myself | 812 (71.2) |
|  | I am disappointed in myself | 239 (21.0) |
|  | I am disgusted with myself | 27 (2.4) |
|  | I hate myself | 62 (5.4) |
| ***Self-Criticalness*** | I don't feel I am any worse than anybody else | 661 (58.0) |
|  | I am critical of myself for my weaknesses or mistakes | 266 (23.3) |
|  | I blame myself all the time for my faults | 140 (12.3) |
|  | I blame myself for everything bad that happens | 73 (6.4) |
| ***Suicidal Thoughts or Wishes*** | I don't have any thoughts of killing myself | 872 (76.5) |
|  | I have thoughts of killing myself, but I would not carry them out | 189 (16.6) |
|  | I would like to kill myself | 32 (2.8) |
|  | I would kill myself if I had the chance | 47 (4.1) |
| **Crying** | I don't cry any more than usual | 673 (59.0) |
|  | I cry more now than I used to | 231 (20.3) |
|  | I cry all the time now | 80 (7.0) |
|  | I used to be able to cry, but now I can't cry even though I want to | 156 (13.7) |
| **Agitation** | I am no more irritated by things than I ever was | 533 )46.8( |
|  | I am slightly more irritated now than usual | 358 (31.4) |
|  | I am quite annoyed or irritated a good deal of the time | 170 (14.9) |
|  | I feel irritated all the time | 79 (6.9) |
| **Loss of interest** | I have not lost interest in other people | 507 (44.5) |
|  | I am less interested in other people than I used to be | 305 (26.8) |
|  | I have lost most of my interest in other people. | 204 (17.9) |
|  | I have lost all of my interest in other people | 124 (10.9) |
| **Indecisiveness** | I make decisions about as well as I ever could | 586 (51.4) |
|  | I put off making decisions more than I used to | 303 (26.6) |
|  | I have greater difficulty in making decisions more than I used to | 187 (16.4) |
|  | I can't make decisions at all anymore | 64 (5.6) |
| **Appearance** | I don't feel that I look any worse than I used to | 907 (79.6) |
| **(Worthlessness)** | I am worried that I am looking old or unattractive | 101 (8.9) |
|  | I feel there are permanent changes in my appearance that make me look unattractive | 86 (7.5) |
|  | I believe that I look ugly | 46 (4.0) |
| **Loss of energy** | I can work about as well as before | 500 (43.9) |
|  | It takes an extra effort to get started at doing something | 376 (33.0) |
|  | I have to push myself very hard to do anything | 217 (19.0) |
|  | I can't do any work at all | 47 (4.1) |
| **Sleeping Pattern** | I can sleep as well as usual | 537 (47.1) |
|  | I don't sleep as well as I used to | 412 (36.1) |
|  | I wake up 1-2 hours earlier than usual and find it hard to get back to sleep | 107 (9.4) |
|  | I wake up several hours earlier than I used to and cannot get back to sleep | 84 (7.4) |
| **Irritability** | I don't get more tired than usual | 538 (47.2) |
|  | I get tired more easily than I used to | 376 (33.0) |
|  | I get tired from doing almost anything | 132 (11.6) |
|  | I am too tired to do anything | 94 (8.2) |
| **Changes in Appetite** | My appetite is no worse than usual | 741 (65.0) |
|  | My appetite is not as good as it used to be | 255 (22.4) |
|  | My appetite is much worse now | 89 (7.8) |
|  | I have no appetite at all anymore | 55 (4.8) |
| **Weight loss** | I haven't lost much weight, if any, lately | 782 (68.6) |
|  | I have lost more than three kilograms | 200 (17.5) |
|  | I have lost more than five kilograms | 61 (5.4) |
|  | I have lost more than eight kilograms | 41 (3.6) |
| **Concentration Difficulty** | I can focus as usual | 462 (40.5) |
|  | I can't focus like usual | 341 (29.9) |
|  | It's hard to stay focused on anything for long | 249 (21.80 |
|  | I can't focus on anything | 88 (7.7) |
